# Report of Cases Treated in the Surgical Wards of the Pennsylvania Hospital, and Discharged between January 22d and February 3d, 1839

**Published:** 1839-02-09

**Authors:** T. Harris

**Affiliations:** Attending Surgeon


### CLINICAL REPORTS.

### PENNSYLVANIA HOSPITAL.

*Report of Cases treated in the Surgical Wards of the Pennsylvania Hospital, and discharged between January 22d and February 3d,* 1839. Dr. T. Harris, Attending Surgeon.

[Reported by H. H. Smith, M. D., Resident Surgeon.]

A case of fracture of the lower end of the fibula, with a partial dislocation of the ankle internally, consequent on the fracture, was admitted December 1st, 1838. The dislocation was reduced, and the foot firmly fastened to the fracture-box, afterwards leeched, &c. Discharged cured, fifty-three days after admission. The date of the discharge was later than usual, owing to extraneous circumstances, but the patient had been able to walk nearly two weeks previously. The treatment of this case, is that usual in the hospital in this kind of accident, and performs the cure always without deformity. The toes being well turned in, and securely fastened to the foot-board, the fracture is generally perfectly reduced—the only modification being the introduction of a pad (such as that placed in the axilla in the apparatus for fracture of the clavicle) on the inner side of the foot, near the ankle, when the fracture is two or three inches above the joint, so as to enable us to turn the foot more inwardly.

A case of fracture of both bones of the leg, near the knee, in a man who had been discharged but ten days, for a fracture of both bones of the same leg, near the lower third, was admitted November 7th, treated by the fracture-box, and discharged January 23d, seventy-seven days after the injury. Violent inflammation of the knee-joint followed this injury, and lengthened the period of cure—the usual period for simple fractures being about fifty-six days. The old fracture was so firm, that no injury resulted to it from the cause producing the last one—a bank falling on the limb.

A case of compound fracture of the leg, with an opening to the knee-joint, caused by a heavy waggon passing over the limb, was admitted February 2d. The patient died February 3d, never having reacted from the exposure consequent on bringing him to the city, a distance of seven miles, without sufficient protection from the cold.

A case of fracture of the upper third of the femur, was admitted November 8th. Dressed with Desault's long splints for eight days; but, owing to a slough on the heel, caused by the extending band, all extension was abandoned. The limb was, therefore, shortened one inch and a half, and the patient discharged February 2d, eighty-six days after the accident. The difficulty of preventing sloughing of the heel from the extending band, is a constant source of deformity in the treatment of fractures of the thigh, especially when erysipelas prevails in the house. Notwithstanding the greatest attention, as shifting the band daily, padding it with cotton, &c., it is sometimes impossible to prevent it.

A case of fracture of the humerus, at the upper third, caused by a blast, was admitted December 19th. An angular splint, extending from the fingers to the arm-pit, was placed on the inside, and three small splints, from the shoulder to the elbow on the other sides of the arm—the angle of the inner one being changed every third day. Discharged cured, January 24th, thirty-six days after the injury.

A case of fracture of the condyles and lower part of the humerus, was admitted December 1st. The patient had been treated in the country for a dislocation of the elbow, for six days, and was at last sent to town, because the dislocation, when reduced, would not remain so: treated by the angular splint, and discharged January 24th, fifty-five days after admission, with some false anchylosis of the joint, which was disappearing under passive motion and frictions.

A case of dislocation of the elbow, backwards and upwards, caused by a fall on the palm of the hand, when the arm was partially flexed: reduced by extension and straightening the arm,—no fracture existed,—afterwards kept at rest on an angular splint. Discharged February 2d, one week after the accident.

